# Critical assessment of synthetic accessibility scores in computer-assisted synthesis planning

**DOI:** 10.1186/s13321-023-00678-z

**Published:** 2023-01-14

**Authors:** Grzegorz Skoraczyński, Mateusz Kitlas, Błażej Miasojedow, Anna Gambin

**Affiliations:** Faculty of Mathematics, Informatics, and Mechanics, University of Warsaw, Stefana Banacha 2, Warsaw, Poland

**Keywords:** Retrosynthesis, Synthetic accessibility scores, Assessment, Computer assisted synthesis planning

## Abstract

Modern computer-assisted synthesis planning tools provide strong support for this problem. However, they are still limited by computational complexity. This limitation may be overcome by scoring the synthetic accessibility as a pre-retrosynthesis heuristic. A wide range of machine learning scoring approaches is available, however, their applicability and correctness were studied to a limited extent. Moreover, there is a lack of critical assessment of synthetic accessibility scores with common test conditions.In the present work, we assess if synthetic accessibility scores can reliably predict the outcomes of retrosynthesis planning. Using a specially prepared compounds database, we examine the outcomes of the retrosynthetic tool AiZynthFinder. We test whether synthetic accessibility scores: SAscore, SYBA, SCScore, and RAscore accurately predict the results of retrosynthesis planning. Furthermore, we investigate if synthetic accessibility scores can speed up retrosynthesis planning by better prioritizing explored partial synthetic routes and thus reducing the size of the search space. For that purpose, we analyze the AiZynthFinder partial solutions search trees, their structure, and complexity parameters, such as the number of nodes, or treewidth.We confirm that synthetic accessibility scores in most cases well discriminate feasible molecules from infeasible ones and can be potential boosters of retrosynthesis planning tools. Moreover, we show the current challenges of designing computer-assisted synthesis planning tools. We conclude that hybrid machine learning and human intuition-based synthetic accessibility scores can efficiently boost the effectiveness of computer-assisted retrosynthesis planning, however, they need to be carefully crafted for retrosynthesis planning algorithms.The source code of this work is publicly available at https://github.com/grzsko/ASAP.

**Supplementary Information:**

The online version contains supplementary material available at 10.1186/s13321-023-00678-z.

## Introduction

**Table 1:** Comparison of analyzed synthetic accessibility scores

	SAscore	SCScore	RAscore	SYBA
Molecule representation	Pipeline Pilot ECFP4 / RDKit Morgan FP radius 2	RDKit Morgan FP radius 2	RDKit Morgan FP radius 2	RDKit Morgan FP radius 2
Training dataset	Molecules from PubChem	Reactions from Reaxys	Molecules from ChEMBL	Molecules from ZINC15
Infeasible training molecules generation	No	No	AiZynthFinder verification	Using Nonpher
Model	Fragment contributions	Neural network	Neural network and GBM	Naïve Bayes

The present era of machine learning (ML) and deep learning (DL) techniques and high computing power provides solutions to problems previously treated as untractable. One of them is computer-assisted synthesis planning (CASP) which consists of two tasks: reactions forward planning and retrosynthesis. The former is predicting the outcomes of reaction for given reactants. The latter is a method of planning the synthesis scheme of chemical compounds from simple precursors available in stock, to synthesized intermediates, and the target molecule. Synthesis planning remained a laborious, manual task until the 1960s when Corey [1] formalized the idea of CASP and then implemented it in LHASA [2] software. Over the years, new solutions were developed that automated subsequent planning elements, required less human intervention, and increased the speed and accuracy of algorithms [3–5]. Over the last decade, several modern, ML-based CASP tools were independently developed: from closed vendor software, e.g. Synthia (previously Chematica) [6, 7], to the closed source with the available interface, e.g. IBM RXN [8], and open-source ones, e.g. LillyMol [9], AiZynthFinder [10–12], ASKCOS Tree-builder [13], AutoSynRoute [14]. Currently, a standard CASP tool [15] consists of three modules: (i) the database of reaction templates and rules on how to apply them to analyzed molecules, (ii) algorithms searching for possible synthetic routes, (iii) a database of in-stock molecules. The aforementioned tools differ significantly in the design of every module. For example, the database of reaction templates may be manually encoded with a rule-based algorithm for reaction prediction, e.g. Synthia. It may be also automatically extracted and reactions may be predicted with a neural network, e.g. LillyMol, AiZynthFinder, and ASKCOS Tree-builder. Finally, reactions may be predicted using a template-free seq2seq algorithm [16] known from natural language processing as implemented in IBM RXN.

Besides CASP tools’ strengths, their key bottleneck is computational complexity. During retrosynthesis planning runtime, potentially exponential in size search space of solution candidates (partial synthetic routes) must be traversed. It makes CASP tools non-applicable when numerous molecules need to be immediately checked for synthesizability. One example is a virtual screening (VS) method known in computer-assisted drug design (CADD). During VS, even billions of compound candidates are evaluated for desired properties; thus, searching for a synthetic route for each of these candidates is computationally intractable.

This limitation may be overcome by scoring the synthetic accessibility, i.e. by predicting how the molecule of a given structure is synthesizable. Previously, synthetic accessibility scores were based on single molecular properties selected manually by experts [17–20]. With the emergence of ML and DL methods, new scores were designed. They can be divided into structure-based and reaction-based approaches. Structure-based approaches evaluate the feasibility of molecular structure, e.g. SAscore [21], SYBA [22], GASA [23]. Reaction-based approaches predict the synthetic accessibility by capturing the similarity of synthetic routes deposited in reaction databases, e.g. SCScore [24], RAscore [25], CMPNN [26], or RetroGNN [27].

Although the majority of these scores are publicly available and documented, their applicability as a pre-retrosynthesis heuristic is known to a limited extent. Moreover, there is a lack of critical assessment of synthetic accessibility scores on the standardized dataset with common test conditions.

In the present work, we assess if synthetic accessibility scores can reliably predict outcomes of retrosynthesis planning. We also analyze if synthetic accessibility scores can speed up the retrosynthesis planning by reducing the size of the search space. Specifically, we analyze the outcomes and runtime of the retrosynthetic tool AiZynthFinder on a specially prepared compounds database. We assess if four scores: SAscore, SCScore, RAscore, and SYBA (cf. Table 1) properly predict the results of retrosynthesis planning and the search complexity. To do this, we analyze the AiZynthFinder partial solutions search trees. Moreover, by in-depth analysis of these search trees, we assess if synthetic accessibility scores can speed up retrosynthesis planning by better prioritizing partial synthetic routes.

To the best of the authors’ knowledge, it is the first of this kind of assessment. Although benchmarks are available in cheminformatics, they focus on the outputs of the CASP tools [28] or on synthetic accessibility scores alone [29, 30]. This assessment is easily reproducible and is designed as a framework for evaluating and comparing novel synthetic accessibility scores. Its source code with usage instructions is publicly available at https://github.com/grzsko/ASAP.

## Methods

**Fig. 1:**
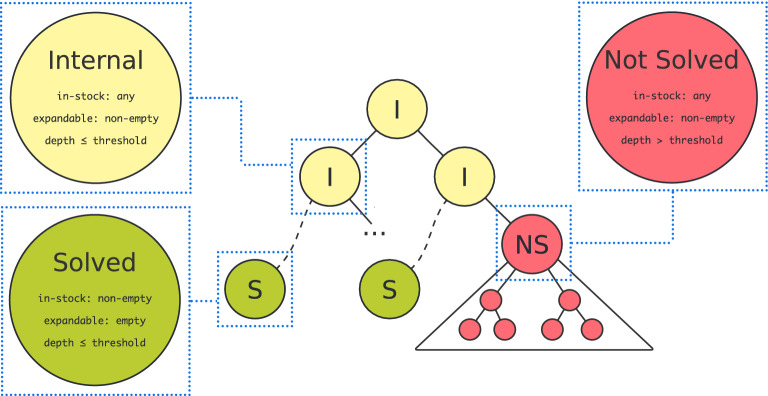
AiZynthFinder search tree nodes classification. Nodes are classified as: internal (I), solved (S), and not solved (NS). Internal nodes have a non-empty list of expandable molecules, but their depth is below a predefined depth. Solved nodes are leaves with all molecules in the in-stock list. A leaf marked as not solved means that it contains at least one expandable molecule and its depth exceeds a predefined threshold. Because we aim to discriminate promising nodes from non-promising ones as early as possible, we define a not solved node as all nodes that have no path to a solved leaf. In the majority of cases, we focus on roots of subtrees of not solved nodes

### Analyzed synthetic accessibility scores

#### SAscore

SAscore [21] is designed as a synthetic accessibility score of drug-like molecules for virtual screening exploration. It is calculated as a sum of fragment scores and complexity penalty. Fragment score is based on statistics of the frequency of Extended Connectivity Fingerprints of diameter 4 (ECFP4) [31] fragments from Pipeline Pilot [32] on almost one million molecules obtained from the PubChem database [33]. ECFP is a method of creating a numeric representation of a chemical structure by traversing it, enumerating atoms, and hashing their representation. The aim of the fragment score is to capture if fragments observed previously in the database are present in the analyzed molecule. The complexity penalty aims to capture if a molecule does not contain too many complex structures to be synthesized. It incorporates among others number of aromatic rings, stereocenters, macrocycles, or the size of the molecule. SAscore achieves values from 1 (easy to synthesize) to 10 (hard to synthesize). It is publicly available in RDKit package [34].

#### SYBA

The idea of the SYBA score is to train a model on comprehensive representations of both existing, easy-to-synthesize compounds as well as non-existing, hard-to-synthesize compounds. The former set was randomized from the ZINC15 database and the latter set was created from an easy-to-synthesize one using Nonpher tool [35] by the iterative perturbing structure of the input molecules (adding/removing of atom or bond) up to a predefined complexity threshold. SYBA is a Bernoulli naïve Bayes classifier trained on both sets. Its implementation is available as a Conda package or at https://github.com/lich-uct/syba.

#### SCScore

SCScore is a score for assessing the molecular complexity expressed as the expected number of reaction steps required to produce a target. This score was trained using neural networks [36] on the set of 12 million reactions obtained from the Reaxys database [37]. Molecules for this score are represented as 1024-bit Morgan fingerprints of radius 2 [38] which are generally similar to ECFP4. It achieves values from 1 (simple molecule) to 5 (complex molecule). This score was used as precursor prioritizer in ASKCOS Tree-builder tool [13] and is publicly available in GitHub repository https://github.com/connorcoley/scscore.

#### RAscore

RAscore is designed as a retrosynthetic accessibility score, i.e. score for fast prescreening molecules for the AiZynthFinder tool. It was trained on over 200000 molecules from ChEMBL [39]. For every molecule, a synthesis route was generated using AiZynthFinder to assess if the molecule is synthesizable. Two models were trained on these outcomes: neural network [36] and gradient boosting machine [40]. RAscore implementation is publicly available at https://github.com/reymond-group/RAscore.

### Analyzed CASP tool

AiZynthFinder is an algorithm for computational synthesis planning. It utilizes the Monte Carlo tree search (MCTS) algorithm [41, 42], which is used for searching the tree of possible partial solutions to the analyzed problem. Here, solutions correspond to synthetic routes of the target molecule. Single MCTS round consists of 4 steps [43]: (1) selection of random leaf node, (2) expansion during which new nodes from leaf are created, (3) rollout, i.e. search simulation from new node till the complete solution or a partial solution exceeding a predefined depth, (4) backpropagation during which nodes are actualized after rollout. The node containing a partial solution is represented by (i) its depth, (ii) the set of in-stock molecules, and (iii) the set of expandable molecules which need to be further transformed into simpler, buyable molecules. Here, the depth of the node is defined as the maximal number of transformations that each of its molecules has to undergo to the target. A leaf node represents a complete solution if it does not need to be expanded, i.e. its list of expandable molecules is empty and its depth does not exceed a predefined threshold. Otherwise, a leaf node represents an infeasible partial solution with a depth exceeding a threshold i.e. it corresponds to the too long synthetic route. The root node of the search tree contains a single expandable molecule representing the target compound. Nodes are connected with directed edges representing a reaction whose product is a single expandable molecule. Leaf selection is made by recursively traversing a search tree starting from the root by selecting children of maximum upper confidence bound (UCB) which expresses current node exploitation and how it is promising:

1 $$\begin{aligned} UCB = \frac{Q}{N_p} + U. \end{aligned}$$

*U* describes how the node was already explored, i.e.

$$\begin{aligned} U = 1.4\cdot \sqrt{2\cdot \frac{\ln N_{-1}}{N_p}}, \end{aligned}$$

where $N_p$ is the number of times the child node has been visited, and $N_{-1}$ is the number of times the parent node has been visited. *Q* describes how the node is promising, i.e. it is a sum of rewards from previous backpropagations. A single reward equals:

2 $$\begin{aligned} 0.95 \cdot \frac{M_{\text {s}}}{M} + 0.05\ \frac{1}{1+\exp (m - 4)}, \end{aligned}$$

where *M* is the number of molecules in the node, $M_{\text {s}}$ is the number of solved molecules and *m* is the maximum number of transformations that every molecule have to undergo to become the root. A reward assesses how molecules of a given node are already expanded and how many steps are used. Nodes are expanded using a neural network applying reaction templates on expandable molecules in the node. Reactions are chosen so that the UCB of the product is maximized.

### Evaluation of synthesis planning and scores

#### Dataset

We prepared a database of 49 compounds. Their detailed list is available in Additional File 1. The majority of these compounds are drugs or plant metabolites, of which 44 have documented synthesis. Molecules in our database were collected to represent various synthesis complexity, starting from easily synthesizable ones such as acetylsalicylic acid, to compounds of known synthesis but the more complex structure, such as morphine, compounds of known low yielding synthesis, such as isocorydine, and not known to be synthesizable. On the other hand, the molecules were collected to represent several examples of high demand for synthesizability, such as drugs, plant metabolites, human metabolites, etc. All compounds have their structure encoded in SMILES notation [44] from PubChem [33] with incorporating stereo orientation. Molecules from this database were further input dataset of AiZynthFinder tool and synthetic accessibility scores for their analysis.

**Fig. 2:**
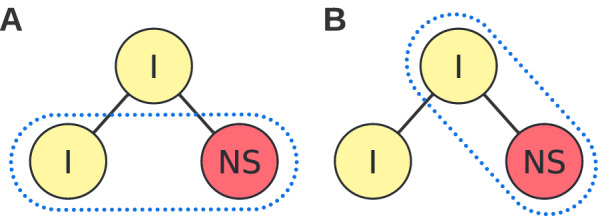
Analyzed nodes configurations Panel **A**: We checked if two nodes, internal and not solved which have the same internal parent can be discriminated by synthetic accessibility scores. Panel **B**: We checked also if synthetic accessibility scores can discriminate internal parents from their not solved children.

#### Analysis of the search trees

In the first analysis, we assessed if synthetic accessibility scores can model and predict outcomes of retrosynthesis planning. To express the complexity of retrosynthesis planning, we analyzed the search trees of AiZynthFinder runtime for molecules from our database. For these trees, we calculated statistics, such as the number of nodes, treewidth, and the number of leaf nodes that are not solved. We omitted to analyze tree depth because AiZynthFinder has strict limits for the depth of the search tree and the results would be uninformative.

Moreover, we checked if synthetic accessibility scores can act as nodes’ prioritization heuristics. To this end, we classified all nodes into three groups: solved, not solved, and internal (cf. Fig. 1). Solved nodes correspond to complete solutions, i. e. all their molecules are available in stock. Not solved nodes correspond to partial solutions of an infeasible synthetic route. We define not solved nodes as nodes for which there is no path leading to a solved node. The rest of the nodes are internal, i.e. nodes having a path to the solved leaf node. They correspond to these partial solutions which eventually lead to a complete solution. For such nodes definition, if the root of a tree is not solved then the algorithm has not found any feasible synthetic route for a given target molecule. We express a score value of a node as one of the statistics (maximum, minimum, arithmetic mean) over all molecules in the node. For making calculations comparable, all scores were transformed so that they achieve values from the range [0, 1] with 0 corresponding to an infeasible (non-synthesizable) molecule and 1 corresponding to a feasible (easily synthesizable) molecule. To check if synthetic accessibility scores properly prioritize nodes, we analyzed if synthetic accessibility scores discriminate internal nodes from not solved ones. Firstly, we considered these pairs connected with a single reaction step. We analyzed two configurations: (i) siblings nodes internal and not solved with internal parent and (ii) internal parent from not solved child (cf. Fig. 2). Secondly, we checked if synthetic accessibility scores correctly discriminate internal nodes from not solved ones in general.

Finally, we checked if modified leaf selection, which incorporates nodes’ synthetic accessibility scores, may speed up retrosynthesis planning. To this point, we modified UCB (Eq. (1)) by substituting a fraction of a reward with one of the synthetic accessibility scores. Specifically, a reward (Eq. (2)) was replaced with the value:

3 $$\begin{aligned} c\cdot \mathcal{S}\mathcal{A} + (0.95-c) \cdot \frac{M_{\text {s}}}{M} + 0.05\ \frac{1}{1+\exp (m - 4)}, \end{aligned}$$

where *c* is a replaced fraction of reward ($\frac{1}{4}\cdot 0.95$, $\frac{2}{4}\cdot 0.95$, $\frac{3}{4}\cdot 0.95$) and $\mathcal{S}\mathcal{A}$ is one of appriopriately transformed synthetic accessibility scores.

## Results and discussion

**Fig. 3:**
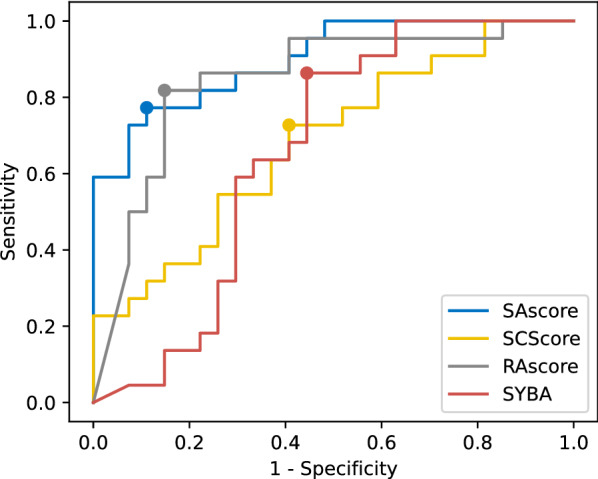
ROC curve for synthetic accessibility scores prediction of AiZynthFinder outcomes. Dots mark the best score threshold. AUCs for curves are listed in Table 2

**Table 2:** Comparison of analyzed synthetic accessibility scores in predicting the AiZynthFinder outcomes

	AUC	Accuracy
SAscore	0.90	0.81
RAscore	0.85	0.85
SCScore	0.67	0.69
SYBA	0.66	0.67

**Fig. 4:**
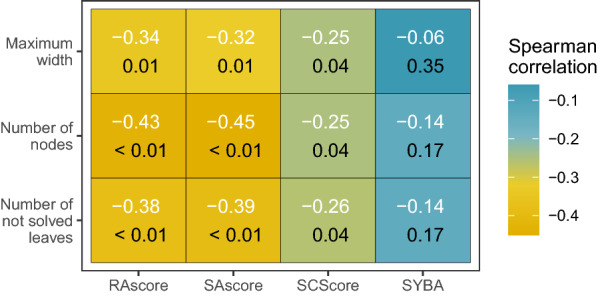
Heatmap of correlation between synthetic accessibility scores and complexity search tree parameters. Colors and white labels indicate the value of the Spearman correlation, black labels indicate the p-value of the correlation test.

**Fig. 5:**
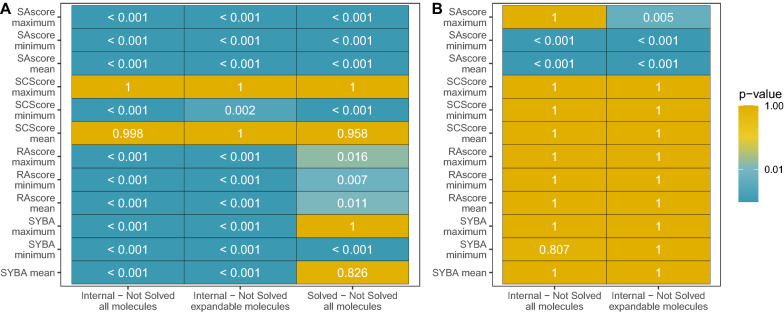
Heatmaps of t-test p-values for hypothesis whether synthetic accessibility scores discriminate node types. Panel **A**: For internal and not solved siblings node pairs and solved and not solved node pairs if their scaled score differences are greater than 0. Panel **B**: For internal parent and not solved child node pairs if their scaled score differences are greater than 0. Here, discrimination between solved and not solved is not applicable.

For all compounds from our database, we performed retrosynthesis planning using AiZynthFinder with default parameters. AiZynthFinder found a synthetic route for 22 compounds. For all found synthetic routes, 20 of them are known (precision 0.91), and for all known synthetic routes, 20 of them are found (sensitivity 0.45).

We assessed if synthetic accessibility scores correctly predicted the results of retrosynthetic planning. To find the optimal score thresholds that discriminate synthesizable target molecules from non-synthesizable ones, we analyzed a receiver operating curve (ROC), cf. Fig. 3. It allows for finding the best balance between the sensitivity and specificity of the classifier. For every score and its optimal threshold, we computed the prediction accuracy of AiZynthFinder’s outcomes. We also measured the quality of scores by calculating the area under the ROC curve (AUC) which describes the probability that a score ranks a randomly chosen synthesizable molecule better than a randomly chosen non-synthesizable molecule. Results are depicted in Table 2. For both AUC and accuracy, SAscore and RAscore achieves high results (AUC and accuracy were both over 0.81). On the contrary, for both SCScore and SYBA, the results are worse by about 20 percentage points. RAscore’s good result is not surprising, because it was trained on the outcomes of the AiZynthFinder algorithm. This, combined with the low sensitivity of AiZynthFinder, allows us to claim that RAscore is a precise heuristic of AiZynthFinder outcomes, but not necessarily a synthetic accessibility score in general. The results of the SAscore may seem surprising. It is a slightly different score from the rest because it is not a standard ML model. It is designed as a combination of scores and penalties derived by experts from the presence of structural fragments in the PubChem database. From this, we infer that in retrosynthesis, human intuition and the power of the human mind still play an important role in planning a synthesis route especially in noticing the irregularities in the general synthesis rules. On the opposite, ML models are prone to imperfections, imbalance, bias, or gaps in training data. This lies in line with recent studies indicating ML limits in cheminformatics, for example for reaction yield prediction [45], for CADD [46], or for graph-based DL models for drug representation [47].

**Fig. 6:**
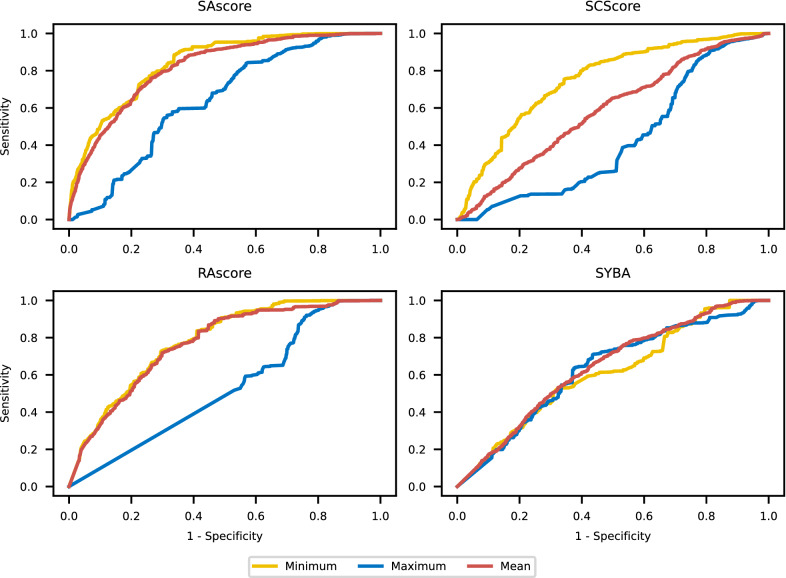
ROC curves for discrimination of internal and not solved nodes by appropriately scaled synthetic accessibility scores. AUCs are depicted in Fig. 7

**Fig. 7:**
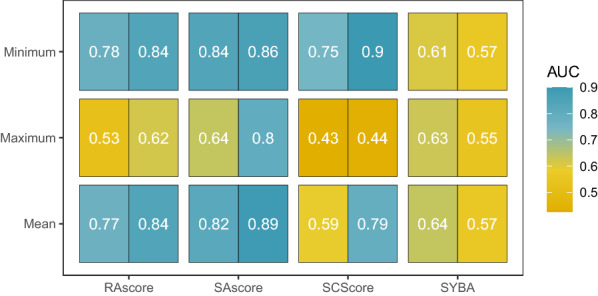
Heatmap of AUC of discrimination between internal and not solved nodes (left) and solved and not solved nodes (right)

We checked also if synthetic accessibility scores can model the complexity of the retrosynthesis planning. We computed a Spearman rank correlation [48] between scores of target compounds and their search tree complexity parameters, such as treewidth, number of nodes, and number of not solved leaf nodes. Results are available in Fig. 4. All of RAscore, SAscore, and SCScore with at least one node aggregating statistic correlate negatively with all complexity parameters with significance below 0.04. On the contrary, SYBA does not correlate with any of the complexity parameters. Analogously as earlier, RAscore and SAscore performed best, the strongest negative correlation was observed between these two scores and the number of nodes.

As a next step, we checked if scores can be a good heuristic for prioritizing nodes corresponding to partial solutions. Well-prioritized nodes would preferably select routes that are more promising for further search and boost the efficiency of retrosynthesis planning. To this end, we checked if synthetic accessibility scores can detect potentially infeasible partial synthesis routes. We assessed this by taking all pairs of internal and not solved siblings nodes and checking if the average score of internal nodes is greater than the score of not solved nodes (cf. Fig. 2A). We used a one-sample t-test [49] for score differences of node pairs. The alternative hypothesis was that the mean of the pair differences distribution is greater than 0. We checked also if incorporating in-stock set molecules would not bias the node statistics. Thus, we repeated the same test on node scores incorporating only expandable molecules. Results are depicted in Fig. 5A. Practically, all scores with at least one aggregating statistic can correctly discriminate internal nodes from not solved and solved nodes from not solved. Omitting the set of in-stock molecules did not change the results.

We repeated the same analysis for pairs of the internal parent node and not solved child (cf. Fig. 2B). Contrary to previous results, only SAscore can significantly discriminate the parent internal node from its not solved child (cf. Fig. 5B).

Moreover, we checked in-depth if synthetic accessibility scores can correctly discriminate internal nodes from not solved ones and solved nodes from not solved ones. We collected all internal, solved, and not solved nodes. To find a threshold properly discriminating nodes, we analyzed ROC curves of synthetic accessibility scores, cf. Fig. 6 and Additional file 2: Figure S1. AUCs are depicted in Fig. 7. Practically, all scores except SYBA correctly discriminate internal nodes from not solved and solved from not solved. Note that for each of the rest of the scores, only the mean and minimum aggregating functions are efficient. It is because minimum detects the presence of non-synthesizable outliers while maximum reports the best synthesizable molecules. Analogously as earlier, SAscore achieved the best results and RAscore was slightly worse. The rest of the scores were considerably worse.

Finally, we analyzed if directly replacing a fraction of the reward with an appropriately scaled synthetic accessibility score may boost the retrosynthesis planning as in Eq. 3. If so then nodes during leaves selection would be better prioritized by UCB and thus computation time decreased. This replacement, however, did not significantly improve any parameter of search tree complexity (cf. Additional file 2: Tables S1–S4). It may be caused by undermined reward fraction in UCB formula (1) or high fitting of search algorithm design to its internal scorings. It should be noticed that the reward function is only used in the backpropagation phase and is calculated by the MTCS procedure in the leaf nodes to update the statistics of the search tree. On the other hand, the molecules at leaf nodes score highly on the synthesizability scale, as they are typically small and often purchasable. Therefore, the alternative approach to guide the selection of nodes would be adding the synthesizability scores to the UCB statistics calculated in the internal nodes. Such modification is worth implementing and we plan to incorporate it in further work.

## Conclusions

In the present work, we analyzed if synthetic accessibility scores can effectively boost the retrosynthesis process. Our analyses consisted of checking if synthetic accessibility scores correctly model retrosynthesis planning outcomes and effectively discriminate feasible partial synthetic routes from infeasible ones. We confirmed that synthetic accessibility scores can in the majority of cases well discriminate feasible molecules from infeasible ones and can be potential boosters of retrosynthesis planning tools.

Today, the big-data era requires retrosynthesis planning tools to be a fast and accurate replacement for laborious, human-mind-based manual work. We show, however, that designing retrosynthesis planning algorithms is still a challenging task and require constant improvement for faster runtime and more accurate results. For example, replacing a fraction of UCB failed to improve AiZynthFinder accuracy which suggests that synthetic accessibility scores need to be carefully crafted for the target tool.

Moreover, high, outlying SAscore results suggest that currently, pure ML techniques still do not replace completely a human mind in the retrosynthesis planning process. This implies that the accuracy of scores, although increasing, is still limited. This results in a constant need for improving the quality of training datasets, because ML models may overfit to specific properties of training datasets that appeared to be unbalanced or biased. Also, there should be constant pressure for better model design. We conclude that hybrid ML and human intuition-based synthetic accessibility scores with carefully crafted retrosynthesis planning algorithms can still efficiently boost the effectiveness of computer-assisted retrosynthesis planning. These tools may help for both finding synthetic routes of newly designed compounds as well as recognizing what is still unknown in chemistry.

## Supplementary Information

**Additional file 1.** A Microsoft Excel spreadsheet containing a database of analyzed molecules with their SMILES encoding and synthesis information.

**Additional file 2: Figure S1.** ROC curves for discrimination of solved and not solved nodes bysynthetic accessibility scores.** Table S1.** Tree max depth for replacing a fraction of the reward with anappropriately scaled synthetic accessibility score (SAscore, SCScore, SYBA).** Table S2.** Tree maximum width for replacing a fraction of the reward with anappropriately scaled synthetic accessibility score (SAscore, SCScore, SYBA).** Table S3.** Tree node count for replacing a fraction of the reward with anappropriately scaled synthetic accessibility score (SAscore, SCScore, SYBA).** Table S4.** Number of not solved leaves for replacing a fraction of the reward withan appropriately scaled synthetic accessibility score (SAscore, SCScore, SYBA).

## Data Availability

Project name: ASAP - Critical Assessment of Synthetic Accessibility scores in computer-assisted synthesis Planning, Project home page: https://github.com/grzsko/ASAP, Operating system(s): Linux or macOS, Programming language: Python 3, Other requirements: Conda package management system, License: MIT, Any restrictions to use by non-academics: none. The molecule dataset being the input of AiZynthFinder is available in Additional file 1.

## Abbreviations

AUC: Area under the ROC curve
CADD: Computer-assisted drug design
CASP: Computer-assisted synthesis planning
DL: Deep learning
ML: Machine learning
MCTS: Monte Carlo tree search
ROC: Receiver operating curve
UCB: Upper confidence bound
VS: Virtual screening
