# Supplementary material for: Critical assessment of synthetic accessibility scores in computer-assisted synthesis planning
Source: J Cheminform. 2023 Jan 14;15:6. doi: 10.1186/s13321-023-00678-z (PMC9840255; doi:10.1186/s13321-023-00678-z)
Supplement: Supplementary file 2 — Additional file 2: Figure S1. ROC curves for discrimination of solved and not solved nodes bysynthetic accessibility scores. Table S1. Tree max depth for replacing a fraction of the reward with anappropriately scaled synthetic accessibility score (SAscore, SCScore, SYBA). Table S2. Tree maximum width for replacing a fraction of the reward with anappropriately scaled synthetic accessibility score (SAscore, SCScore, SYBA). Table S3. Tree node count for replacing a fraction of the reward with anappropriately scaled synthetic accessibility score (SAscore, SCScore, SYBA). Table S4. Number of not solved leaves for replacing a fraction of the reward withan appropriately scaled synthetic accessibility score (SAscore, SCScore, SYBA). [file 13321_2023_678_MOESM2_ESM.pdf]

Supplementary material
to article *Critical assessment of synthetic accessibility scores in computer-assisted synthesis planning.*

Grzegorz Skoraczyński, Mateusz Kitlas, Błażej Miasojedow, Anna Gambin

Supplementary Figures

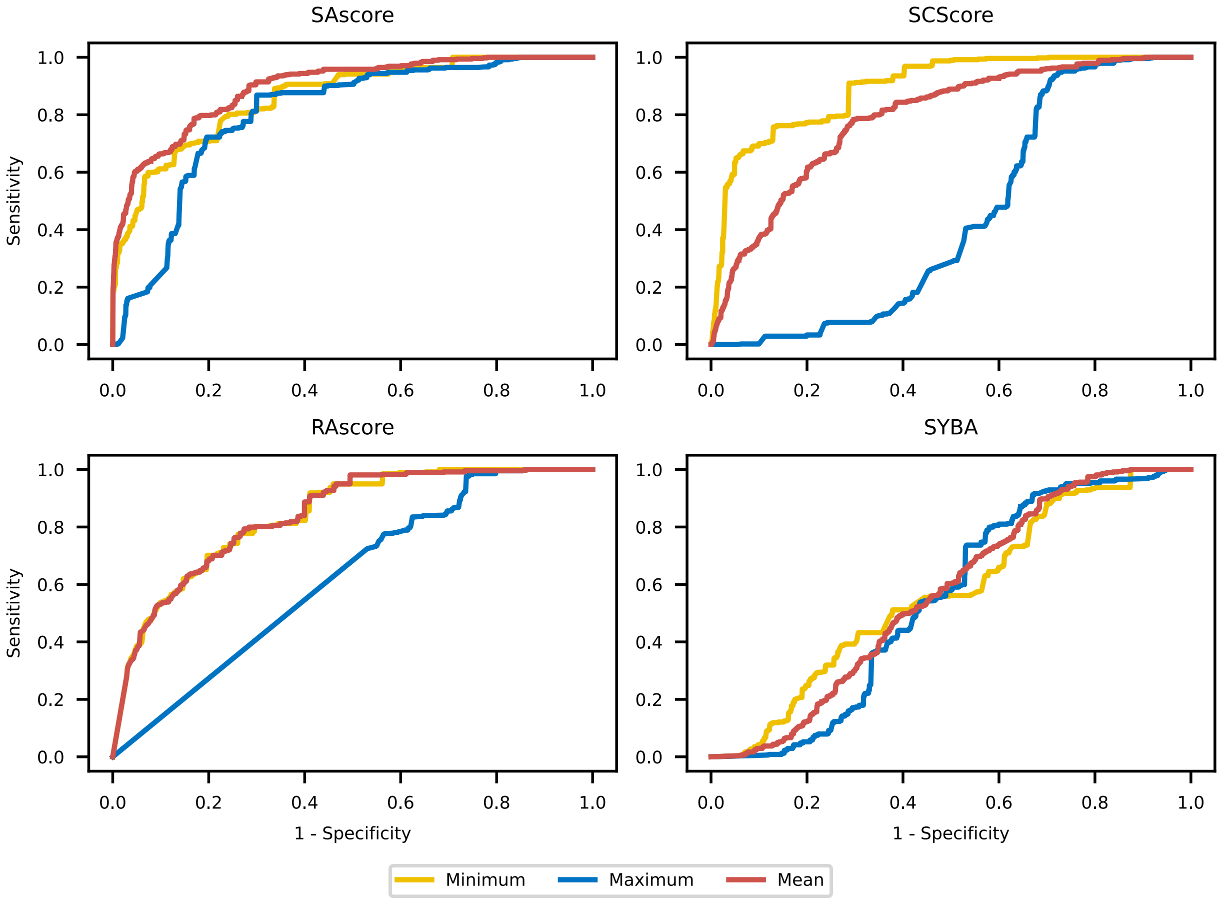

Supplementary Figure S 1 ROC curves for discrimination of solved and not solved nodes by synthetic accessibility scores.

Supplementary Tables

	Score	Exchanged score fraction	Std deviation	Mean	Median
**0**	-	0	2.966002	8.755102	9.0
**1**	SCScore minimum	0.2375	2.984759	8.775510	9.0
**2**	SCScore maximum	0.2375	3.016959	8.857143	9.0
**3**	SCScore minimum	0.4750	3.114369	8.877551	9.0
**4**	SCScore maximum	0.4750	2.948397	8.795918	9.0
**5**	SCScore minimum	0.7125	3.016821	8.795918	9.0
**6**	SCScore maximum	0.7125	3.026056	8.836735	9.0
**7**	SAscore minimum	0.2375	3.030320	8.795918	9.0
**8**	SAscore maximum	0.2375	2.950374	8.775510	9.0
**9**	SAscore minimum	0.4750	3.079537	8.836735	9.0
**10**	SAscore maximum	0.4750	2.903415	8.755102	9.0
**11**	SAscore minimum	0.7125	3.046222	8.836735	9.0
**12**	SAscore maximum	0.7125	3.001180	8.816327	9.0
**13**	SYBA minimum	0.2375	2.952209	8.755102	9.0
**14**	SYBA maximum	0.2375	2.994372	8.816327	9.0
**15**	SYBA minimum	0.4750	2.976095	8.857143	9.0
**16**	SYBA maximum	0.4750	2.941467	8.795918	9.0
**17**	SYBA minimum	0.7125	2.998959	8.836735	9.0
**18**	SYBA maximum	0.7125	3.037047	8.795918	9.0

Supplementary Table S 1 Tree max depth for replacing a fraction of the reward with an appropriately scaled synthetic accessibility score (SAscore, SCScore, SYBA).

	Score	Exchanged score fraction	Std deviation	Mean	Median
**0**	-	0	79.605493	115.836735	104.0
**1**	SCScore minimum	0.2375	72.193571	112.918367	102.0
**2**	SCScore maximum	0.2375	92.332363	121.000000	106.0
**3**	SCScore minimum	0.4750	70.729288	115.979592	108.0
**4**	SCScore maximum	0.4750	79.498344	119.183673	108.0
**5**	SCScore minimum	0.7125	78.982919	119.326531	109.0
**6**	SCScore maximum	0.7125	71.927795	116.673469	109.0
**7**	SAscore minimum	0.2375	77.377965	118.448980	108.0
**8**	SAscore maximum	0.2375	80.494308	119.183673	106.0
**9**	SAscore minimum	0.4750	78.892469	119.244898	109.0
**10**	SAscore maximum	0.4750	81.146876	120.204082	107.0
**11**	SAscore minimum	0.7125	79.059502	119.408163	108.0
**12**	SAscore maximum	0.7125	78.432918	119.306122	107.0
**13**	SYBA minimum	0.2375	67.852180	114.714286	108.0
**14**	SYBA maximum	0.2375	70.466778	115.673469	108.0
**15**	SYBA minimum	0.4750	79.456316	119.000000	108.0
**16**	SYBA maximum	0.4750	81.474173	120.285714	109.0
**17**	SYBA minimum	0.7125	93.411559	123.489796	109.0
**18**	SYBA maximum	0.7125	79.713763	120.040816	108.0

Supplementary Table S 2 Tree maximum width for replacing a fraction of the reward with an appropriately scaled synthetic accessibility score (SAscore, SCScore, SYBA).

	Score	Exchanged score fraction	Std deviation	Mean	Median
**0**	-	0	383.891782	624.448980	622.0
**1**	SCScore minimum	0.2375	357.194631	614.530612	616.0
**2**	SCScore maximum	0.2375	385.142319	641.918367	646.0
**3**	SCScore minimum	0.4750	362.632884	640.408163	650.0
**4**	SCScore maximum	0.4750	373.379975	645.081633	649.0
**5**	SCScore minimum	0.7125	360.175180	644.408163	649.0
**6**	SCScore maximum	0.7125	368.898435	645.836735	650.0
**7**	SAscore minimum	0.2375	373.173272	639.510204	645.0
**8**	SAscore maximum	0.2375	385.174569	644.979592	649.0
**9**	SAscore minimum	0.4750	372.952424	643.571429	650.0
**10**	SAscore maximum	0.4750	388.082908	650.938776	649.0
**11**	SAscore minimum	0.7125	364.409662	645.081633	640.0
**12**	SAscore maximum	0.7125	363.200205	644.244898	650.0
**13**	SYBA minimum	0.2375	366.721196	636.183673	638.0
**14**	SYBA maximum	0.2375	365.604941	636.081633	651.0
**15**	SYBA minimum	0.4750	369.442529	640.816327	638.0
**16**	SYBA maximum	0.4750	388.511178	649.591837	648.0
**17**	SYBA minimum	0.7125	379.724067	651.571429	650.0
**18**	SYBA maximum	0.7125	361.971850	644.367347	650.0

Supplementary Table S 3 Tree node count for replacing a fraction of the reward with an appropriately scaled synthetic accessibility score (SAscore, SCScore, SYBA).

	Score	Exchanged score fraction	Std deviation	Mean	Median
0	-	0	286.580406	223.551020	132.0
1	SCScore minimum	0.2375	244.677409	211.836735	134.0
2	SCScore maximum	0.2375	295.157197	230.326531	136.0
3	SCScore minimum	0.4750	260.086613	223.122449	136.0
4	SCScore maximum	0.4750	279.672345	229.306122	138.0
5	SCScore minimum	0.7125	263.093437	225.081633	140.0
6	SCScore maximum	0.7125	265.976406	227.020408	141.0
7	SAscore minimum	0.2375	268.964581	225.183673	137.0
8	SAscore maximum	0.2375	287.977563	230.673469	136.0
9	SAscore minimum	0.4750	273.662586	227.183673	138.0
10	SAscore maximum	0.4750	298.367672	234.448980	138.0
11	SAscore minimum	0.7125	262.503722	225.571429	137.0
12	SAscore maximum	0.7125	264.755605	225.979592	139.0
13	SYBA minimum	0.2375	263.764404	223.632653	140.0
14	SYBA maximum	0.2375	258.578303	222.469388	141.0
15	SYBA minimum	0.4750	269.348709	226.387755	135.0
16	SYBA maximum	0.4750	294.816401	232.938776	143.0
17	SYBA minimum	0.7125	286.012455	233.346939	137.0
18	SYBA maximum	0.7125	258.676774	224.142857	139.0

Supplementary Table S 4 Number of not solved leaves for replacing a fraction of the reward with an appropriately scaled synthetic accessibility score (SAscore, SCScore, SYBA).
